# Machine learning for acute kidney injury: Changing the traditional disease prediction mode

**DOI:** 10.3389/fmed.2023.1050255

**Published:** 2023-02-03

**Authors:** Xiang Yu, Yuwei Ji, Mengjie Huang, Zhe Feng

**Affiliations:** Department of Nephrology, Chinese PLA General Hospital, Chinese PLA Institute of Nephrology, State Key Laboratory of Kidney Diseases, National Clinical Research Center of Kidney Diseases, Chinese PLA General Hospital, Beijing, China

**Keywords:** AKI, inpatient, artificial intelligence, machine learning, predictive model

## Abstract

Acute kidney injury (AKI) is a serious clinical comorbidity with clear short-term and long-term prognostic implications for inpatients. The diversity of risk factors for AKI has been recognized in previous studies, and a series of predictive models have been developed using traditional statistical methods in conjunction with its preventability, but they have failed to meet the expectations in limited clinical applications, the rapid spread of electronic health records and artificial intelligence machine learning technology has brought new hope for the construction of AKI prediction models. In this article, we systematically review the definition and classification of machine learning methods, modeling ideas and evaluation methods, and the characteristics and current status of modeling studies. According to the modeling objectives, we subdivided them into critical care medical setting models, all medical environment models, special surgery models, special disease models, and special nephrotoxin exposure models. As the first review article to comprehensively summarize and analyze machine learning prediction models for AKI, we aim to objectively describe the advantages and disadvantages of machine learning approaches to modeling, and help other researchers more quickly and intuitively understand the current status of modeling research, inspire ideas and learn from experience, so as to guide and stimulate more research and more in-depth exploration in the future, which will ultimately provide greater help to improve the overall status of AKI diagnosis and treatment.

## 1. Introduction

AKI is a clinical emergency associated with a variety of acute and chronic comorbidities; even mild AKI may lead to chronic kidney disease, and severe or recurrent events may lead to end-stage renal disease and are strongly associated with an increased risk of death and impaired quality of life (1). With the increasing incidence and mortality of AKI worldwide, clinicians are paying increasing attention to AKI (2).

It is well established that AKI usually occurs in susceptible populations with high-risk factors or following certain specific medical procedures, and several previous studies have obtained more consistent conclusions on the evaluation of risk factors by analyzing the static and dynamic characteristics of a large number of AKI patients (3–8). Based on the preventability of AKI and the ease of diagnosis, increasing research is focused on the early diagnosis of AKI, with the aim of early assessment of inpatients to determine their risk stratification, dynamic adjustment of treatment protocols, replacement of potentially dangerous medical orders, and interventions to avoid or reduce potential kidney injury and ultimately to achieve inpatient renal protection by reducing the incidence of AKI, avoiding a series of subsequent adverse events.

The construction of predictive models for AKI has been an outstanding achievement for nephrologists in the 21st century, and over the past 10–20 years, predictive models have been reported, especially in recent years, the electronic medical record information systems of different medical institutions around the world have gradually improved, the use of electronic health records to build models has become another breakthrough in this field, and since the introduction of artificial intelligence, it has provided more methods for efficient utilization of massive medical data in the age of big data, making mining massive information and training models based on machine learning algorithms become a frontier hotspot. It is exciting to see that the technology has not disappointed in terms of medical decision-making behavior; at least in current studies, such models can predict AKI with an AUC of 0.80 or more, and in some studies, the AUC even reached 0.93. In this report, we set the search keywords as “machine learning” and “acute kidney injury” through the pubmed database, we screened the relevant literature by reviewing the abstract information, after carefully reading the included literature, we focus on reviewing recent advances in the application of machine learning systems to predict AKI risk, providing evidence and summarizing ideas for subsequent studies to be conducted.

## 2. Concept of machine learning

Machine learning is a major branch of AI technology, defined as the study of algorithms that use computer systems to learn from sample data and past experience (9). The popular machine learning algorithms include supervised learning and unsupervised learning, among which supervised learning includes decision trees, support vector machines, naive Bayes, k-nearest neighbor, logistic regression, random forests, gradient boosting trees, generalized additive models, artificial neural networks, and integrated tree models; unsupervised learning includes principal component analysis and k-mean classification. Among them, neural networks are a new addition to the field, and the most representative, deep learning, is considered to be the closest to the original goal of AI, solving many complex pattern recognition challenges. Other techniques include fully connected neural networks, convolutional neural networks (CNNs), recurrent neural networks (RNNs), generative adversarial networks (GANs), and deep reinforcement learning (10–12) (Table 1).

**Table 1:** Description of machine learning algorithms.

**Concept**	**Type**	**Description**	**Advantages**	**Disadvantages**
**Supervised learning**	**Supervised learning is a computational method where we give the algorithm a dataset and given the correct answer, the machine goes through the data to learn the correct answer**.			
Linear regression	Regression	■ The simplest regression method that uses a linear equation (y = m ^*^ x + b) to model the dataset.	Fast modeling speedSimple calculation variable interpretation can be provided based on the coefficients	It is necessary to first determine whether the relationship between variables is linear Does not fit non-linear data well
Logistic regression	Classification	■ Logistic regression is an algorithm that estimates the probability of an event based on one or more inputs and is more commonly used in classification problems.	■ Less time-consuming and faster classification calculation ■ Intuitive observation sample probability scores ■ Not affected by multicollinearity and can be combined with L2 regularization to solve the problem ■ Low computational cost, easy to understand and implement	■ Computational performance degrades when the feature space is large ■ Easy to underfit, generally not very accurate ■ Does not handle large number of class features well ■ Conversion is required for non-linear features
Naive Bayes	Classification	■ A probabilistic classifier, which is a classification method based on Bayes' theorem and the assumption of the conditional independence of features.	■ Stable classification efficiency ■ Fast speed for large volume training and queries ■ Performs well on small data sizes, can handle multiple classification tasks, and is suitable for incremental training ■ Less sensitive to missing data and simpler algorithms	■ Need to calculate the probability prior ■ High error rate in classification decisions ■ Sensitive to the form of expression of the input data
Decision trees	Classification	■ An algorithm for solving classification problems. The decision tree algorithm uses a tree structure and uses layers of inference to achieve the final classification.	■ Decision trees are easy to understand and interpret and can be analyzed visually ■ Can handle both nominal and numeric data ■ More suitable for handling samples with missing attributes ■ Able to handle unrelated features ■ Runs relatively fast when testing datasets ■ Provides reliable and effective results for large data sources in a relatively short period of time	■ Prone to overfitting ■ Easy to ignore the interconnectedness of attributes in a dataset ■ For data with inconsistent sample sizes in each category, different decision criteria lead to different attribute selection tendencies when attribute classification is performed by decision trees
Random forest	Classification	■ The random forest method is an integrated learning method containing multiple decision trees for classification, regression and other tasks.	■ High-dimensional (many features) data can be computed without dimensionality reduction and without feature selection ■ The importance of features can be judged ■ The interaction between different features can be judged ■ Not easily overfitted ■ Training is faster, and it is easy to use parallel methods ■ Can balance the error of unbalanced datasets ■ Accuracy can be maintained if a large portion of the features are missing	■ Overfitting on some noisy classification or regression problems ■ For data with attributes that have different values, attributes with more value divisions will have a greater impact on the random forest
Support vector machines	Classification	■ Support vector machines is a class of generalized linear classifiers that perform binary classification of data in a supervised learning manner, and whose decision boundary is the maximum margin hyperplane solved for the learned samples.	■ Can solve high-dimensional problems, i.e., large feature spaces ■ Able to handle interactions of non-linear features ■ No local minimal value problem ■ Stronger generalization ability	■ Not very efficient when the observation sample is large ■ There is no universal solution for non-linear problems, and sometimes it is difficult to find a suitable kernel function ■ Weak explanatory power for high-dimensional mapping of kernel functions, especially radial basis functions ■ Conventional algorithms only support binary classification
Gradient boosting decision tree	Classification	■ Boosted trees use additive models and forward stepwise algorithms to implement the optimization process of learning, which is also one of the integrated learning methods.	■ High accuracy can be obtained with relatively little tuning time ■ Flexible handling of various types of data, including continuous and discrete values, for a wide range of uses ■ Some robust loss functions can be used, which are more robust to outliers	■ Dependencies between weak learners make it difficult to train data in parallel
Adaptive boosting	Classification	■ Adaptive boosting (AdaBoost) belongs to one of the boosting categories in the ensemble method. It is a binary classification model. As an iterative algorithm, the core idea is to train different classifiers (weak classifiers) for the same	■ Flexible in the use of various regression classification models to build weak learners ■ The implementation of AdaBoost in Sklearn is based on a weighted learning perspective, which is simple and easy to understand ■ Controls the number of iterations to prevent overfitting to some extent	■ Sensitive to anomalous samples, which may receive higher weights in the iterations, affecting the final prediction accuracy
		training set, and then aggregate these weak classifiers to form a stronger final classifier (strong classifier).		
Extreme gradient boosting	Classification/Regression	■ Extreme gradient boosting (XGBoost) is also a type of integration algorithm as a boosted tree model, which is a combination of many tree models together to form a very strong classifier. In addition, the tree model used is the CART regression tree model.	■ Compared to other machine learning libraries, users can easily use XGBoost and obtain satisfactory results ■ Fast and effective in processing large-scale datasets and does not require large amounts of hardware resources such as memory ■ Compared to deep learning models, the effect is similar without fine-tuning parameters ■ XGBoost internally implements a boosted tree model, which can automatically handle missing values	■ Compared with the deep learning model, it is unable to model spatiotemporal location and capture high-dimensional data such as image, voice and text well ■ Deep learning is far more accurate than XGBoost when it has a large amount of training data and can find a suitable deep learning model
Light gradient boosting machine	Classification/Regression	■ Light gradient boosting machine (LightGBM) is a fast, distributed, high-performance gradient boosting framework based on decision tree algorithms. It can be used for sorting, classification, regression and many other machine learning tasks.	■ Faster training speed and higher efficiency ■ Lower memory footprint ■ Higher accuracy than any other enhancement algorithm ■ Compared to XGBoost, it is also capable of handling big data due to its reduced training time ■ Supports parallel learning	■ The computation process may grow deeper decision trees, thus creating overfitting ■ Since LightGBM is a bias-based algorithm, it is more sensitive to noise ■ In finding the optimal solution, it is based on the optimal cut variables and does not take into account the idea that the optimal solution is a combination of all features
Categorical boosting	Classification/Regression	■ Categorical boosting (CatBoost) is a GBDT framework with fewer parameters, support for categorical variables and high accuracy based on oblivious trees as the base learner implementation.	■ Can rival any advanced machine learning algorithm in terms of performance ■ Reduces the need for much hyperparameter tuning and reduces the chance of overfitting, which also makes the model more generalizable ■ Can handle categorical and numerical features and supports custom loss functions	■ The processing of category-based features requires a great deal of memory and time ■ The setting of different random numbers has certain influences on the prediction results of the model
Generalized additive model	Classification	■ The generalized additive model is an interpretable model that uses the sum of the unary and binary shape functions of the predictor variables to explain the response variables.	■ Non-linear functions can be introduced ■ Because it is “additive”, the hypothesis testing method of the linear model can still be used	■ Because of the “additive” assumption, important interactions may be missing from the model and can only be compensated for by manually adding interaction terms
K-nearest neighbor	Classification/Regression	■ The core idea of the algorithm is that if a sample belongs to a class in which most of the k most adjacent samples in the feature space belong to that class, then that sample also belongs to that class and has the characteristics of the samples in that class.	■ The theory is mature and the idea is simple and can be used for both classification and regression ■ Can be used for non-linear classification ■ No assumptions about the data, has high accuracy, and ■ is an online technique where new data can be added directly to the dataset without retraining	■ The treatment of unbalanced samples is less effective, and the prediction bias is relatively large ■ Requires a lot of memory ■ More computationally intensive for datasets with large sample sizes ■ Each time the classification is performed again, a global operation is performed, which is computationally intensive ■ There is no theory for the choice of k-value size, which is often chosen in conjunction with k-fold cross-validation to obtain the optimal k-value
Artificial neural networks	Classification/Regression	■ Artificial neural networks are broadly similar to unusually complex networks composed of neurons, which are individual units connected to each other, and each unit has a numerical amount of inputs and outputs, which can be in the form of real numbers or linear combinatorial functions.	■ High accuracy of classification ■ High parallel distribution processing capability ■ Distributed storage and high learning capacity ■ Strong robustness and fault tolerance to noisy nerves ■ Able to fully approximate complex non-linear relationships with associative memory function	■ Neural networks require a large number of parameters, such as network topology, initial values of weights and thresholds ■ The inability to observe the internal learning process and the difficulty in interpreting the output can affect the credibility and acceptability of the results ■ The study time is too long and may not even achieve the purpose of learning
**Unsupervised learning**	**In unsupervised learning, there is no “right answer” for a given dataset, all data are the same. The task of unsupervised learning is to uncover the underlying structure from a given dataset**.			
Principal component analysis	Classification	■ A set of potentially correlated variables is transformed into a set of linearly uncorrelated	■ Reduces the computational overhead of the algorithm	■ Eigenvalue decomposition has some limitations, e.g., the transformed matrix must be a square matrix
		variables by an orthogonal transformation, and the transformed set of variables is called the principal component.	■ Noise removal ■ Makes the results easy to understand ■ Completely parameter free	■ In the case of a non-Gaussian distribution, the resulting principal element may not be optimal
K-means clustering	Classification	■ K-means clustering is a technique for clustering data into a specified number of classes to reveal the intrinsic properties and patterns of the data.	■ For large datasets, k-means clustering is efficient ■ The computational complexity is close to linear	■ The algorithm is affected by the initial values and outlier points, and the results are unstable every time; usually the results are not the global optimal solution but the local optimal solution ■ Cannot solve the case of relatively large differences in data cluster distribution very well ■ Not truly applicable to discrete data ■ K-value based on artificial selection lacks objectivity
**Other special learning algorithms**	**Reinforcement learning is closer to the nature of biological learning and therefore promises higher intelligence. It is concerned with how an intelligent body can adopt a set of behaviors in its environment in order to obtain the maximum cumulative reward**.			
	**Deep learning is the process of learning the intrinsic laws and levels of the representation of sample data, and the information obtained from these learning processes can be of great help in the interpretation of data such as text, images and sound**.			
Reinforcement learning	Classification	■ Reinforcement learning is used to describe and address the problem of learning strategies by which an intelligent body learns to maximize reward or achieve a specific goal during its interaction with the environment.	■ Ability to model sequential decision problems ■ Training does not require labeled data ■ There are good theoretical guarantees, and the main algorithms have corresponding convergence proofs	■ Feedback is delayed, not generated immediately ■ Delayed reward
Deep learning	Classification	■ The concept of deep learning originates from the study of artificial neural networks, and a multilayer perceptron with multiple hidden layers is a deep learning structure that mainly includes convolutional neural networks, deep neural networks and recurrent neural networks.	■ Strong learning ability ■ Wide coverage and good adaptability ■ Data-driven, high ceiling ■ Excellent portability	■ Application scenarios that can only provide a limited amount of data do not allow deep learning algorithms to provide an unbiased estimation of the patterns of the data ■ To achieve good accuracy, big data support is needed ■ As the complexity of graph models in deep learning leads to a dramatic increase in the time complexity of the algorithm, higher parallel programming skills and more and better hardware support are needed to ensure the real-time performance of the algorithm

## 3. Machine learning modeling ideas

**Figure 1:**
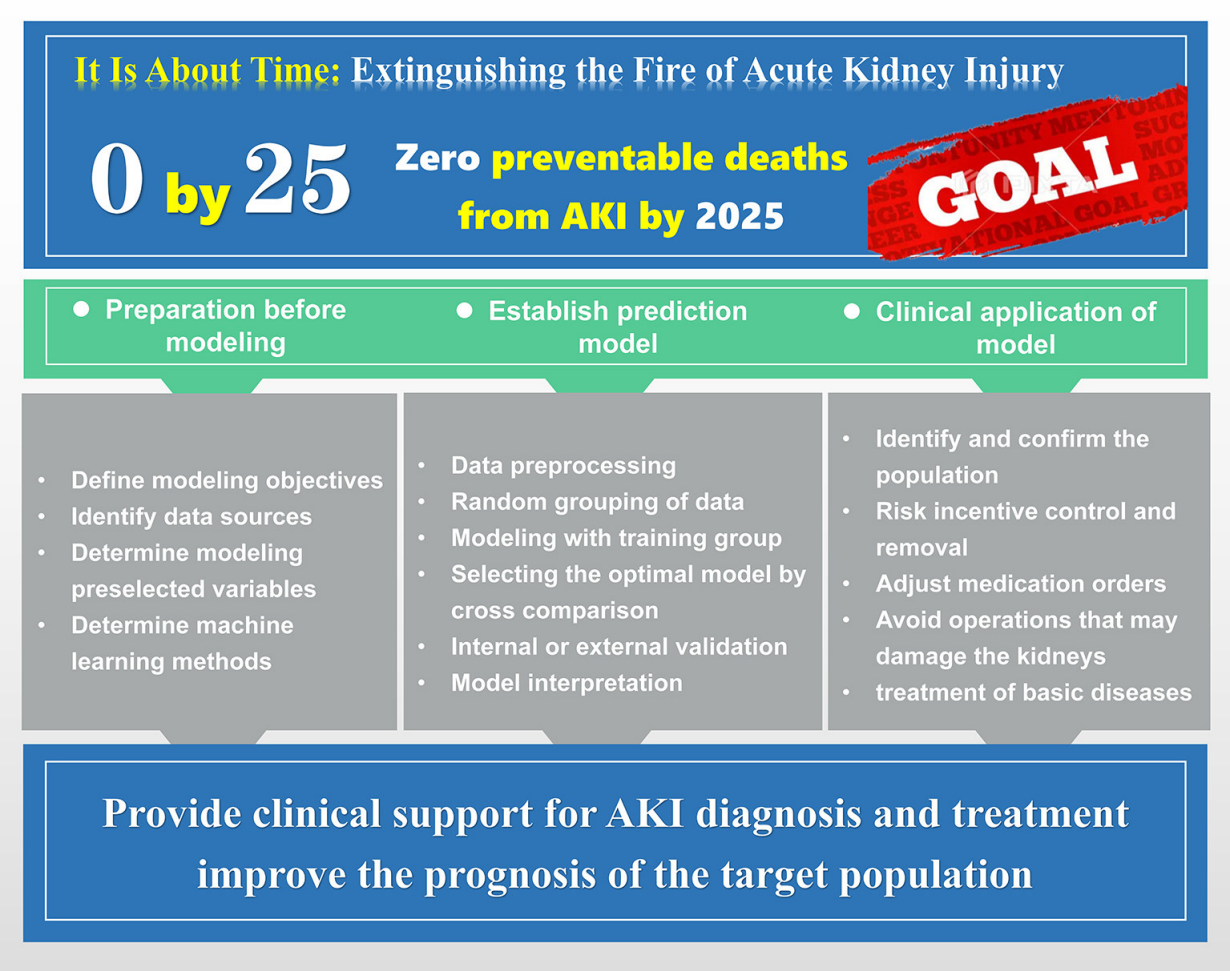
The modeling idea and flow chart of machine learning prediction model. Including the preparation work before modeling, the specific process of model training, and the applicability of the final model to the patient population.

The process of machine learning modeling is continuous and consists of the following steps based on summarized research experiences. **Step 1: Data acquisition**. The research team defines the data concept according to the modeling objectives and accesses the authorized data, including structured data or unstructured data, using different data repositories from multiple medical institutions. Even though EMR data are now standardized through coding systems, this process is still tedious and time-consuming. **Step 2: Data preparation**. The collected data must be cleaned, managed and organized into a more computable format. This includes the labeling of supervised learning data, the transformation of categorical values, and the processing of outliers, missing values, and extremely imbalanced data. **Step 3: Feature selection**. A feature is the “column name” of the data, such as drug n, respiratory rate, or age. The model development process relies on feature selection from a large feature base, including LASSO or the Breiman and Cutler random forest method, and this process is repeated until the researcher is satisfied with the model performance. **Step 4: Model training**. Based on a training dataset of fixed ratio classification according to the model type, preselected features are used for iterative training. This process can be performed by replacing features and model configurations to continuously optimize model hyperparameters, fine-tune model performance, and reduce prediction errors. **Step 5: Model validation**. This step includes two substeps of internal and external verification. The preferred test method is k-fold cross-validation, where internal validation can identify data overfitting or underfitting during model training, while external validation can check the model performance under real conditions. **Step 6: Model trial**. The model is employed in a health care environment with new data to test its predictive feasibility and reliability in routine clinical work and clinician acceptability. **Step 7: Model evaluation and interpretation**. Model evaluation is an important process to judge the usability and reliability of models and provides key metrics for parallel comparisons among models. The evaluation of machine learning prediction models is similar to that of diagnostic experiments in that the main reference index is the area under the receiver operating characteristic curve (AUROC). In addition, other indices are often cited as auxiliary evaluations in studies, including accuracy, recall, precision, sensitivity, specificity, positive predictive value, negative predictive value, F1-value, etc. Effective model interpretation can help clinicians better understand the results of the model output and the relationship between variables. At present, the SHAP method based on the algorithmic game theory is widely used. which can interpret the prediction results from both global and local perspectives, and is proved to be more consistent with human intuition than the existing methods. **Step 8: Model monitoring and updating**. After the model is formally used, each step of its operation is monitored to ensure the expected use and prediction accuracy while continuously updating and optimizing the model performance through new data performance. It is important to note that the above processes and steps are not necessarily continuous and unidirectional, allowing for feedback during the process, with the exception of the deep learning process (Figure 1).

## 4. Machine learning prediction model for AKI

### 4.1. AKI prediction model for critical care settings

In 2015, the International Society of Nephrology proposed the “AKI 0 by 25” initiative to achieve zero deaths from AKI by 2025, and the ICU, as the most affected area with high morbidity and mortality from AKI, is the greatest obstacle to achieving this goal (13). Against this background, there is a general consensus to identify the risk of AKI among critically ill patients early and to take a more proactive role in AKI prevention and management, it is worth mentioning that these measures are highly time dependent and time sensitive.

In recent years, there has been an abundance of research on machine learning prediction models for AKI for critically ill patients, and there are several features of this type of research. First, the choice of model training data was mostly focused on three publicly available databases, MIMIC-III, AmsterdamUMCdb and eICU (14–25). The use of local databases is not common, and only information from the Mayo Clinic and SHZJU-ICU can be retrieved (21, 26, 27). Public databases are highly integrated and easy to access, but it is inevitable that some of the missing information affects the authenticity of the data; for example, the variables with a large proportion of missing values in the MIMIC-III database include the lowest albumin level (74.1%), the highest bilirubin level (67.2%), the highest lactate level (55.8%), the highest C-reactive protein level (99.0%), the highest aspartate aminotransferase level (66.8%), the highest pH level (36.6%) and the lowest base excess level (64.8%) (14). Moreover, these studies mainly included European and American populations, and the generalizability of the models is doubtful for Asian populations. In addition, the differences in data extraction criteria settings or data preprocessing methods lead to a certain degree of incomparability among studies from the same database sources. Second, of the 14 studies we retrieved, only 3 studies conducted external validation of the model (20, 21, 26). While this is crucial for model generalization and relies on external validation for optimization of model hyperparameters, which is well understood by researchers, there are various constraints to external validation, including non-uniformity of case data formats across centers, self-protection of some medical structures for data security, or difficulty in matching predictions from other centers due to the complexity of the model. Third, all models were able to achieve the above moderate discrimination of AKI events with AUC values ranging from 0.69 to 0.926, incorporating both traditional modeling methods, such as logistic regression, in individual studies (14–16, 18, 20–24). In some studies that also incorporated traditional modeling methods, such as logistic regression, or compared with physicians' subjective diagnoses, machine learning modeling was also simultaneously superior. Fourth, interpretability is very important in the medical field, and a medically assisted diagnostic system must be understandable and interpretable; ideally, it should be able to explain the complete logic of providing corresponding decisions to all relevant parties to gain the trust of physicians, but the process of achieving model interpretation in the construction of predictive models regarding AKI for critically ill patients is rare, which somehow has led to a disconnect between modeling and analysis, forgoing additional analysis of AKI characteristics and risk factors and wasting the potential for the effective use of large amounts of information (19, 22, 26). Fifth, while variable screening for modeling is necessary and critical, the inclusion of static and dynamic variables reflects different modeling objectives. Most models include both at the same time so that patients with high dimensional variables can reference foundation conditions, such as demographic information and the basic state of complications. It is also possible to take into account objective indicators such as vital signs and laboratory tests in real time and to dynamically track the risk trajectory, which can be combined with the gradual or sudden onset of AKI to better predict AKI reliably. On the other hand, 57% of patients meet AKI criteria on the first day of ICU admission, according to the study results (27). Therefore, some studies have included only prehospital data, including basic information and admission diagnosis, to achieve early prediction of early-onset AKI (Table 2).

**Table 2:** AKI prediction model for critical care settings.

**References**	**Modeling data sources**	**Data volume**	**Model performance AUC value**		**External verification**			**Machine learning methods**	**Model explanation**	**Diagnostic criteria**
					**Data source**	**Data volume**	**Model performance AUC values**			
Shawwa et al. (26)	Mayo Clinic	98,472	0.69		MIMIC-III	51,801	0.656	Gradient boosting model	Yes	KDIGO: Creatinine and urine volume
Li et al. (16)	MIMIC-III	14,470	0.779		None			Naive Bayes Support vector machines Logistic regression Random forest Gradient boosting decision tree	None	KDIGO: Creatinine and urine volume
Zimmerman et al. (14)	MIMIC-III	23,950	0.783		None			Logistic regression Random forest Artificial neural networks	None	KDIGO: Creatinine
Zhang et al. (20)	MIMIC-III	2,395	0.88		The First Affiliated Hospital of Fujian Medical University, China	499	0.78	Extreme gradient boosting Adaptive boosting Random forest Logistic regression Multilayer perceptron	None	KDIGO: Creatinine
Liang et al. (21)	SHZJU- ICU and MIMIC-III	58,492	0.86		AmsterdamUMCdb	15,341	0.86	Multiple logistic regression Random forest Extreme gradient boosting Adaptive boosting Light gradient boosting machine Gradient boosting decision tree	None	KDIGO: Creatinine and urine volume
Sun et al. (15)	MIMIC-III	14,469	0.83		None			Logistic regression Random forest Naive Bayes Support vector machines	None	KDIGO: Creatinine
Alfieri et al. (23)	eICU and MIMIC-III	35,573	0.89		None			Deep learning Logistic regression	None	AKIN: Creatinine and urine output
Qian et al. (18)	MIMIC-III	17,205	0.905		None			Logistic regression Support vector machines Random forest Extreme gradient boosting Light gradient boosting machine Convolutional neural networks	None	KDIGO: Creatinine and urine output
Wei et al. (19)	MIMIC-III	25,711	0.926		None			Extreme gradient boosting Logistic regression	Yes	KDIGO: Creatinine and urine output
Fujarski et al. (22)	AmsterdamUMCdb	23,106	0.883		None			Categorical boosting Support vector machines		KDIGO: Creatinine and urine output
Sato et al. (17)	eICU	AKI I	5,342	0.742	None			Convolutional neural networks	None	KDIGO: Creatinine
		AKI II	1,450	0.844						
Le et al. (25)	MIMIC-III	12,347	0.86		None			Convolutional neural networks Extreme gradient boosting	None	KDIGO: Creatinine
Parreco et al. (24)	eICU	151,098	0.834 ± 0.006		None			Gradient boosting decision tree Logistic regression Deep learning	None	KDIGO: Creatinine

AUC, Area Under Curve; MIMIC-III, Medical Information Mart for Intensive Care III; AmsterdamUMCdb, The Amsterdam University Medical Centers Database; eICU, eICU Collaborative Research Database; SHZJU- ICU, The Second Affiliated Hospital of ZheJiang University School of Medicine, China; LightGBM, Light gradient boosting machine.KDIGO, Kidney Disease: Improving Global Outcomes; AKIN, Acute Kidney Injury Network.

### 4.2. AKI prediction model for all care settings

Although it is very beneficial to build predictive models for critically ill patients, the risk of AKI among general inpatients should not be taken lightly. Since the beginning of machine learning modeling research, researchers have been diligently trying to build a predictive model for the entire inpatient population to achieve large predictive coverage once and for all to maximize the benefits of research. Subsequent studies have proven that these ideas are feasible, and the results were also reliable.

At present, this kind of model is mainly divided into two types, namely, “one-time” prediction models and real-time prediction Models. In 2015, Cronin et al. (28) included more than 1.6 million hospitalization data points of veterans for model training, and the results suggested that the AUC values of the three models for predicting AKI during 1–3 periods were 0.746–0.785, 0.714–0.720, and 0.823–0.825, respectively, but surprisingly, the traditional logistic regression and LASSO regression models performed slightly better than the random forest model, this may be due to the variable selection function of LASSO regression model, which can reduce overfitting by eliminating unimportant interfering variables, thus effectively improving model performance. In the 2018 study by Koyner et al. (29), which included ICU, general ward and emergency patients, care instruments such as the Morse fall scale were included in the analysis of variables for the first time, and the study focused on reflecting longitudinal data over time using a discrete time survival analysis framework, with the final gradient boosting model predicting AUC values of 0.9 and 0.87 for AKI 24 and 48 h in advance, respectively. In addition, the authors explored the creation of an algorithmic model that did not include changes in creatinine, suggesting that excluding this factor did not affect the model's ability to differentiate AKI independent of baseline renal function levels. In 2019, Tomasev et al.'s (30) team conducted a collaborative study with Deepmind, a Google company, whose inclusion of source data was consistent with earlier studies by Cronin et al. (28) with similarly large amounts of data (more than 700,000 adult case data, about 6 billion data points and 600,000 record features) and whose model developed using deep learning recurrent neural networks predicted AUC values over 0.9 for AKI events 48 h in advance, this algorithms has been proved to be very suitable for processing high-frequency time series data. In addition, researchers used ablation analysis to identify many factors related to the risk of AKI, which may explain why it was difficult for researchers to analyze this risk in the past.

Some studies have also restricted the age of the included subjects; for example, Kate et al. (31) developed a model for older adults over 60 years of age with a high incidence of AKI, in which four methods were utilized to model both AKI prediction and detection analysis, and the results suggested that the AUC values for both were 0.621–0.644 and 0.692–0.743, respectively, indicating that the detection of AKI is easier than the prediction. In addition, comorbidities were found to be more significant in predicting AKI in ablation experiments, including previous history of AKI and respiratory failure (Table 3).

**Table 3:** AKI prediction model for all care settings.

**Reference**	**Modeling data sources**	**Data volume**	**Model performance AUC value**		**External verification**				**Machine learning methods**	**Diagnostic criteria**
					**Data source**	**Data volume**	**Model performance (AUROC)**			
Koyner et al. (29)	University of Chicago Hospital, US	121,158	24 h in advance	0.90	No				Gradient boosting machine	KDIGO: Creatinine
			48 h in advance	0.87						
			Dialysis 48 h in advance	0.96						
Mohamadlou et al. (32)	Stanford Medical Center, US	48,582	Onset of illness	0.872	MIMIC-III	19,737	Onset of illness	0.841	Boosting decision trees	NHS-AKI: Creatinine
			12 h in advance	0.800			12 h in advance	0.749		
			24 h in advance	0.795			24 h in advance	0.758		
			48 h in advance	0.761			48 h in advance	0.707		
			72 h in advance	0.728			72 h in advance	0.674		
He et al. (33)	University of Kansas Health System—KUHS	96,590	24 h in advance	0.744	No				Logistic regression Random forest	KDIGO: Creatinine
			Any time	0.734						
			Day 1 after admission	0.764						
			Day 2 after admission	0.764						
Kate et al. (31)	Aurora Health Care (15) hospitals	25,521		0.743	No				Logistic regression Support vector machines Decision trees Naive Bayes	AKIN: Creatinine
Cronin et al. (28)	US Department of Veterans Affairs	1,620,898	AKI I	0.746–0.758	No				Logistc regression LASSO regression Random forest	KDIGO: Creatinine
			AKI II	0.714–0.720						
			Dialysis	0.823–0.825						
Churpek et al. (34)	University of Chicago (UC)	48,463	48 h in advance AKI II	0.86	NorthShore University Health System (NUS)	246,895		0.86	Gradient boosting machine	KDIGO: Creatinine
					Loyola University Medical Center (LUMC)	200,613		0.85		
Kim et al. (35)	Seoul National University Bundang Hospital, South Korea	69,081	AKI I	0.88	Seoul National University Hospital, South Korea	72,352	AKI I	0.84	Recurrent neural network Extreme gradient boosting	KDIGO: Creatinine
			AKI II	0.93			AKI II	0.90		
Cheng et al. (36)	University of Kansas Medical Center - KUMC	48,955	0.76		No				Logistic regression Random forest Adaptive boosting	KDIGO: Creatinine
Tomasev et al. (30)	US Department of Veterans Affairs	703,782	Any AKI	0.921	No				Recurrent neural network	KDIGO: Creatinine
			AKI II III	0.957						
			AKI III	0.980						

KDIGO, Kidney Disease: Improving Global Outcomes; AKIN, Acute Kidney Injury Network. NHS, National Health Service; MIMIC-III, Medical Information Mart for Intensive Care III.

Building continuous real-time prediction models is also a goal pursued by researchers, and in a study by Kim et al. (35) in 2021, a continuous prediction model was developed for general inpatients based on various dynamic and static clinical features using recurrent neural network algorithms, in order to achieve real-time prediction of AKI risk. Its applicability to support clinical decision-making was demonstrated by external validation, with internal and external validation AUC values of 0.88 and 0.84 for any stage of AKI and 0.93 and 0.90 for patients with stage 2 AKI or higher, respectively, both of which were better than XGBoost model, again proving the effectiveness of recurrent neural network algorithm in processing sequence data (Table 3).

The applicability of models that are constructed based on all inpatients to critically ill patients, such as ICU inpatients, is an important criterion for evaluating the performance of the model and one of the objectives for the construction of this kind of model. In 2018, a study by Mohamadlou et al. (32) that relied on Stanford local data modeled using the boosting decision tree method set and externally validated with the MIMIC-III database suggested that the model showed higher prediction values across different datasets in different windows (at AKI onset and 12, 24, 48, and 72 h before onset) and showed higher AUC values than SOFA scores. In another study by Churpek et al. (34) in which the above Koyner et al. (29) model was simplified by reducing the number of preselected variables, followed by a large multicenter study of nearly 500,000 inpatients in three health systems and six hospitals, the AUC value for predicting at least stage 2 AKI within 48 h was 0.86 in the internal validation cohort, while in the two external validation cohorts, the values were 0.85 and 0.86 (Table 3).

Some researchers have doubts about several modeling strategies mentioned above, mainly in the setting of the time range of data collection and using this to conduct a multiclinical perspective study in the hope of reflecting the clinical application of the models more realistically. He et al. (33) focused on answering this question in their study in 2019, which evaluated the performance of the models in differentiating AKI by setting different prediction time windows, and found that the AUC values for all models ranged from 0.720 to 0.764, which first confirmed the advantages of the strategy based on machine learning methods for modeling, and that the best model performance was achieved by predicting AKI one day in advance, which is precisely the most commonly used modeling strategy today. A similar study by Cheng et al. (36) was conducted to address the same two questions: how to predict the development of AKI in hospitalized patients early and accurately, and how to assess whether preadmission data enhanced model performance. The study used different data collection time windows for multiple datasets with three kinds of modeling methods for comparison. The results suggested that the AUC values of the random forest model with the best performance that predicted AKI 1–3 days in advance were 0.765, 0.733, and 0.709, respectively, and that the AUC values did not change significantly after adding preadmission data compared with post-admission data only (Table 3).

### 4.3. Special surgery-related AKI prediction model

Given this background, there is an increasing amount of research on cardiac surgery-associated AKI (CSA-AKI) machine learning prediction models, which are able to capture AKI signals flexibly and effectively compared with traditional risk scoring methods while revealing more clearly the complex interconnections between CSA-AKI and its associated factors and are more applicable to the multifactorial pathogenic hypothesis of CSA-AKI. In the first CSA-AKI machine learning modeling study carried out by Lee et al. (37) in 2018, multiple machine learning approaches had stronger predictive efficacy than the previous eight risk scoring models (risk scoring system AUC values clustered at 0.55), and their poor performance may be due to the low number of predictors and the lack of intraoperative non-linear variables, such as transfusion volume or hemodynamic changes, which often represent acute intraoperative responses. Regarding the analysis of the contribution of variables at different stages of the perioperative period, in the study by Tseng et al. (38) in 2020, the importance matrix and SHAP summary plots of the random forest were used to provide double the evidence; that is, more than half of the top 20 important features were intraoperative features, which meant that intraoperative variables were the main influence on early renal function decline after cardiac surgery, which proved the value of intraoperative data. These data reflect the intraoperative acute physiological reactions associated with the prediction of CSA-AKI, whereas prediction models reported in previous studies have placed more emphasis on preoperative status. However, this has also been questioned by studies such as the most recent study by Petrosyan et al. (39) in 2022, in which a hybrid machine learning approach was used for the first time to derive and validate a model to predict CSA-AKI of any stage using only preoperative variables, which not only had outstanding performance compared to traditional logistic regression and other single machine learning approaches to modeling but was also able to adapt to the correlation between multiple variables and prevent overfitting of the data. In the study by Zhang et al. (40) in 2022, the types of procedures included were consistent with Lee and Tseng's study, both including patients undergoing coronary artery bypass grafting and valve replacement, and the modeling methods also overlapped; however, the modeling variables included preoperative, intraoperative, and early post-operative variables, such as early post-operative intubation, PaO2/FiO2 ratio, hemoglobin, serum potassium, and lactate dehydrogenase, and the AUC values of the models of these various machine learning methods were 0.857–0.881. Similarly, in another study conducted by Li et al. (41) in 2020, patients undergoing cardiac aortic surgery were added to the training set, and the variables included preoperative and intraoperative factors as well as post-operative central venous pressure, resulting in an AUC value of 0.845 for the prediction of severe AKI by the Bayes network model (Table 4).

**Table 4:** Special surgery-related AKI prediction model.

**Reference**	**Modeling data sources**	**Data volume**	**Model performance** **AUC value**		**Type of surgery**	**Machine learning methods**	**Model explanation**	**Diagnostic criteria**
Lei et al. (42)	Fuwai Hospital in Beijing, China	897	0.80		Aortic surgery	Logistic regression Support vector machines Random forest Gradient boosting	None	KDIGO: Creatinine
Penny-Dimri et al. (43)	The Australian and New Zealand Society of Cardiac and Thoracic Surgeons (ANZSCTS) database	97,964	0.77–0.78		Aortic surgery, cardiopulmonary diversion surgery, valve surgery, constrictive pericarditis surgery	Logistic regression Gradient boosted machine K-nearest neighbor L neural networks	Yes	Improvement criteria
Zhang et al. (40)	Nanjing First Hospital, China	1,457	0.857–0.881		Coronary artery bypass grafting, valve surgery	Extreme gradient boosting Random forest Deep forest Logistic regression	Yes	KDIGO: Creatinine
Li et al. (41)	Zhongshan Hospital, Fudan University, Shanghai, China	3,639	AKI	0.755	Valve surgery Coronary artery bypass grafting aorta + valve + CABG, valve + great vessel	Bayesian networks	None	KDIGO: Creatinine and urine volume
			Severe AKI	0.845				
Lee et al. (44)	Seoul National University Hospital	2010	0.78		Coronary artery bypass grafting valve surgery	Decision trees Random forest Extreme gradient boosting Support vector machines Neural networks Deep learning	None	KDIGO: Creatinine
Tseng et al. (38)	Far Eastern Memorial Hospital (FEMH), New Taipei City	671	0.839		Coronary artery bypass grafting valve surgery, combination of both treatments	Logistic regression Support vector machines Random forest Extreme gradient boosting Integration algorithm (RF + XGBoost)	Yes	KDIGO: Creatinine
Petrosyan et al. (39)	Cardiocore, University of Ottawa Heart Institute	6,522	0.74		Cardiopulmonary diversion surgery	Hybrid algorithm (Random forest + logistic regression) Logistic regression Enhanced logistic regression	None	KDIGO: Creatinine
Lee et al. (45)	Seoul National University Bundang Hospital	4,104	0.81		Unilateral partial or total nephrectomy	Support vector machines Random forest Extreme gradient boosting Light gradient boosting machine	None	KDIGO: Creatinine
Lazebnik et al. (46)	Cancer Institute, University College London, UK	723	0.75		Open partial nephrectomy	Random forest	None	RIFLE and AKIN: Creatinine
Zhu et al. (47)	Peking University First Hospital, China	87	0.749		Isolated partial nephrectomy	Decision trees Random forest Logistic regression Support vector machines Extreme gradient boosting	None	KDIGO: Creatinine
Bredt et al. (48)		145	0.81		Deceased donor liver transplantation	Logistic regression Artificial neural networks	None	KDIGO: Creatinine
Lee et al. (44)	Seoul National University Hospital, South Korea	1,211	0.90		Deceased donor/living donor liver transplantation	Decision trees Random forest Gradient boosted machine Support vector machines Naive Bayes Multilayer perceptron Deep belief networks	None	AKIN: Creatinine
Dong et al. (49)	Changzheng Hospital, Shanghai, China	2,450	0.92		Liver cancer resection	Logistic regression Support vector machines Random forest Extreme gradient boosting Decision trees	None	KDIGO: Creatinine
He et al. (50)	The First Affiliated Hospital of Zhejiang University School of Medicine, China	493	0.85		Cardiac death donor liver transplantation	Random forest Support vector machines Decision trees Conditional reasoning tree Logistic regression	None	KDIGO: Creatinine and urine volume
Lei et al. (42)	The First Affiliated Hospital of Zhengzhou University, Zhengzhou, China	1,173	0.772		Liver cancer resection	Gradient boosting decision tree Random forest Decision trees	None	KDIGO: Creatinine
Ko et al. (51)	Seoul National University College of Medicine, Seoul National University Bundang Hospital, South Korea	5,302	0.78		Knee arthroplasty	Gradient enhancement	None	KDIGO: Creatinine
Nikkinen et al. (52)	Oulu University Hospital, Oulu, Finland	648	0.91/0.98		Knee arthroplasty, hip arthroplasty	RUSBoost Naive Bayes Support vector machines	None	KDIGO: Creatinine and urine output

KDIGO, Kidney Disease: Improving Global Outcomes; AKIN, Acute Kidney Injury Network; RIFLE, Risk, Injury, Failure, Loss of renal function and End-stage renal disease.

The incidence of CSA-AKI also varies by type of cardiac surgery, and it is currently believed that cardiac major vascular surgery leads to a higher rate due to extracorporeal circulation. For example, Lei et al. (42) reported in their study on CSA-AKI modeling of patients undergoing major vascular surgery in 2020 that up to 72.6% of patients in the training cohort developed varying degrees of CSA-AKI. Based on this cohort modeling, the random forest method also achieved an AUC value of 0.8, much higher than logistic regression and other models. In the Penny-Dimri et al. (43) 2021 study, not only was the data volume of the training cohort large but the included objects were also more abundant, except for aortic surgery, valve surgery and cardiopulmonary bypass surgery, and included some patients with constrictive pericarditis surgery. Based on gradient boosting, k-nearest neighbor and neural network methods, the AUC values of multiple models ranged from 0.77 to 0.78, although they were lower than in previous study results; considering the heterogeneity of the training objects, there is even more reason to believe that the models are generalizable. The introduction of Shapley values for model interpretation is rare and critical in all studies, as it more intuitively illustrates the details of the contribution of the model variables and is more likely to gain the confidence of clinicians (Table 4).

Over the past few decades, liver transplantation has become the treatment of choice for patients with end-stage liver disease (ESLD), and advances in organ preservation, surgical approaches, anesthetic techniques, and immunosuppressive therapy have substantially improved expected outcomes, but recent data still report a 4–94% incidence of AKI after liver transplantation from both living and deceased donors, with 8–17% of patients requiring renal replacement therapy (53). In the few studies currently available, the authors used multiple machine learning methods for modeling comparisons, with optimal AUC values of 0.81, 0.85, and 0.90 in a single study, which were all superior to the values of logistic regression models for same-group comparisons (44, 48, 50). However, due to differences in modeling ideas, there are also differences in the categories of preselected variables and variable contribution characteristics of models, as in the latest study by Luis Cesar Bredt et al. (48) in 2022, where the main included variables in the modeling study with an artificial neural network approach were CKD, MELD score, intraoperative arterial hypotension, massive blood transfusion and extended criteria donor. In the study by Lee et al. (44) in 2018, it was proven that the XGBoost model had the best performance, and the top five variables that were ultimately included were cold ischemia time, mean venous oxygen saturation, mean cardiac index, urine output, and preoperative blood glucose. Both of the abovementioned models were dominated by preoperative and intraoperative variables, but there was almost no overlap in the comparison of variable importance. On the one hand, this may be related to the modeling sample size and functional adaptability of different machine learning algorithms; on the other hand, it may be associated with the source of the liver donor, such as in the latter study of simultaneous living and deceased donor transplantation patients. Certainly, current modeling studies are not interested in post-operative data to achieve early prediction and to prevent implications for post-operative interventions and prevention, even though some studies have confirmed that certain post-operative factors are equally risk factors for AKI, including immunosuppressive drugs, infectious agents, antibiotics, sepsis, long-term hypotension, and the use of radiographic contrast agents (54) (Table 4).

Hepatectomy is one of the most important treatments for primary liver cancer, and the incidence of AKI after hepatectomy (LSA-AKI) has been reported to be 0.9–17.9% (53), but AKI events are often underestimated in clinical practice. Many studies have investigated the risk factors associated with LSA-AKI, and several classical AKI scoring systems, such as the Kalisvaart score and Park score, have been established; however, due to the multifactorial nature of LSA-AKI, such risk scores are inefficient in predicting the occurrence of AKI (55, 56). There are few studies on machine learning modeling of LSA-AKI. In the study by Dong et al. (49), clinical data from 2,450 patients were retrospectively analyzed to compare the model performance of multiple machine learning algorithms, and by incorporating preoperative and intraoperative data, including age, cholesterol, time of surgery, serum creatinine and platelet count, the random forest model had an AUC value of 0.92. Another study by Lei et al. (57) also included preoperative and intraoperative data, and the results suggested that the decision tree model performed even better, with an AUC of 0.722. Unlike a previous study, this study also confirmed that tumor size was a significant predictor. Although the results of these two studies are different, the model performance of the latter study is significantly inferior to that of the former by parallel comparison, and the machine learning model performs significantly better than the traditional logistic regression model. However, the current study still has room for improvement; borrowing from Dong et al. (49), the model development should be more focused on the combination of novel biomarkers and model interpretation and should also consider the accuracy and recall rate to ensure model reliability (Table 4).

Previous studies have focused on long-term changes in renal function after nephrectomy, and models for predicting post-operative AKI are rare, especially in modeling studies using machine learning methods. In 2021, in a study by Lee et al. (45), a variety of machine learning methods were used for modeling comparison, and the results showed that the LightGBM model had the best predictive performance, with an AUC value of 0.81, much higher than that of the logistic regression model. Similarly, in a study by Lazebnik et al. (46) in 2022, the random forest model also showed better predictive performance with an AUC value of 0.75. However, in another study performed in 2020 by Zhu et al. (47), by incorporating preoperative, intraoperative and post-operative variables and using multiple methods to learn and train information on 87 patients, the results showed that the best performing XGBoost model had an AUC value of 0.749, which was lower than that of the classical logistic regression model of 0.826. It may be difficult to conduct parallel comparisons among the only three studies at present because of the degree of variation in the between-group design; for example, there was a certain proportion of RN patients among the 4,104 patients included in the first study (45), whereas the third study included all patients with isolated kidney (47). In addition, in a single study, differences in operator and surgical approaches (manual or robotic, laparoscopic or open) may also lead to confounding bias, making the accurate prediction of outcomes complex and difficult. However, there is relative consistency in the evidence of similar studies, including the inclusion analysis of modeling variables more in favor of type of surgery, male sex, tumor size, age, operation time, intraoperative ischemia time, and renal score, which is generally consistent with the results of previous risk factor studies. Additionally, the evaluation of the efficacy of the machine learning methods was positive, even though the results of the third study were slightly different. However, the reasons for this were related to the small amount of modeling data, which did not ensure that the machine learning method could fully exploit the information to establish the relationship between variables (47). In conclusion, similar to other types of AKI, the application of large-scale data information and machine learning algorithms for the prediction of AKI after nephrectomy has been shown to be feasible, even though none of the findings can be directly generalized across centers (Table 4).

Although there has been little previous interest in AKI after orthopedic surgery, studies have suggested that the incidence of AKI after undergoing hip and knee arthroplasty ranges from 0.5 to 24.0% (58–60), therefore post-arthroplasty AKI can be considered another example of a clinical prediction that could benefit from machine learning. In 2022, Ko et al. (51) used gradient boosting modeling to obtain internally and externally validated AUC values of 0.78 and 0.89 in a modeling study of patients undergoing total knee arthroplasty. In the same year, Nikkinen et al. (52) included patients undergoing knee and hip replacement and compared the models based on creatinine and urine volume, respectively, and the results suggested that the AUC values of the two models were 0.91 and 0.98, respectively. Although there were differences in sample size, machine learning methods, and case characteristics between the two studies, both obtained acceptable results, while in terms of variable selection, both agreed that preoperative creatinine level, male sex, ASA classification, and age were important risk factors for AKI, and this result was largely consistent with previous studies. However, the greatest contribution of similar studies is likely to be the emphasis on post-operative AKI in orthopedic surgery and the introduction of machine learning methods to provide new ideas for AKI prediction studies after orthopedic surgery, especially arthroplasty (Table 4).

### 4.4. Specific disease-related AKI prediction models

Sepsis is the most common cause of AKI in critically ill patients, and its incidence increases with the severity of sepsis, while mortality is significantly higher among SA-AKI patients than among non-AKI patients (61), therefore, identifying patients at risk for AKI is crucial for the management of patients with sepsis. Based on the complexity of the pathogenesis of SA-AKI, it is difficult to realize the early prediction of SA-AKI in clinical work. Currently, the performance of new biomarkers and some AKI scoring systems is not satisfactory. Similarly, the utilization of machine learning methods for SA-AKI is extremely rare, and there was only one relevant study by Yue et al. (62). In this study, data from 3,176 patients were included for model training, and the results showed that the AUC value of the XGBoost model was 0.817, which showed good predictive accuracy in terms of discrimination and calibration while outperforming previous scoring systems and traditional modeling methods. Even though the results do not equate to usefulness in clinical practice as stated by the authors, this study is the first of its kind in SA-AKI research, and while promising for the early prediction of SA-AKI, it is also worthwhile to attribute risk factors for morbidity by ranking the importance of variables such as urine volume, mechanical ventilation, body mass index, eGFR, lowest sCr, and minimum BUN. The study is also meaningful to future parallel studies. The focus remains on increasing the dimensionality of variables and screening for variable characteristics that contribute more to model performance, including comorbidities such as diabetes, hypertension and cardiovascular disease, information on the source of infection such as abdominal infection, the nature of pathogens such as Gram-negative bacteria, invasive operations such as mechanical ventilation, and pharmacological interventions such as diuretics and ACEIs/ARBs (Table 5).

**Table 5:** Specific disease-related AKI prediction models.

**Reference**	**Modeling data sources**	**Data volume**	**Model performance** **AUC value**		**Comorbidity type**	**Machine learning methods**	**Model explanation**	**Diagnostic criteria**
Yue et al. (62)	MIMIC-III	3,176	0.817		Sepsis	Logistic regression K-nearest neighbor Support vector machines Decision trees Random forest Extreme gradient boosting Artificial neural networks	None	KDIGO: Creatinine and urine volume
Qu et al. (63)	Jinling Hospital, Nanjing, China	324	0.919		Acute pancreatitis	Support vector machines Random forest Classification regression tree Extreme gradient boosting Logistic regression	None	KDIGO: Creatinine and urine volume
Yang et al. (64)	Gezhouba Central Hospital of Sinopharm and Xianning Central Hospital, China	424	0.902		Acute pancreatitis	Random forest Support vector machines Extreme gradient boosting Artificial neural networks Decision trees	None	KDIGO: Creatinine and urine volume
Zhang et al. (65)	Zhongshan Hospital, Fudan University, Shanghai, China	6,846	0.822/0.850		Liver cancer Gallbladder cancer	Extreme gradient boosting LASSO regression	None	KDIGO: Creatinine
Scanlon et al. (66)	The Christie NHS Foundation Trust, UK	48,865	30 days in advance	0.881	All types of solid tumors	Random forest	None	NHS-AKI: Creatinine
			1 day in advance	0.947				
Park et al. (67)	Korea Central Cancer Registry (KCCR) in Seoul National University Hospital	21,022	Precision	0.7892	Respiratory tract cancer Gastrointestinal tract cancer Thymus cancer Hematologic malignancy Breast cancer Female genitourinary organ cancer, etc.	Linear regression Ridge regression LASSO regression Least-angle regression Stochastic gradient descent Random forest Multivariate adaptive regression splines	None	KDIGO: Creatinine
			Recall	0.7506				
			F value	0.7576				
Li et al. (68)	Zhongshan Hospital, Fudan University, Shanghai, China	6,459	0.823		Esophageal cancer/Stomach cancer/Intestinal cancer	Bayesian network Naive Bayes Decision trees Logistic regression Random forest Support vector machines	Yes	KDIGO: Creatinine
Li et al. (69)	Zhongshan Hospital, Fudan University, Shanghai, China	2,395	0.835		Lymphoma/Leukemia/Multiple myeloma	Bayesian network Logistic regression	Yes	KDIGO: Creatinine
Tang et al. (70)	Severely burned patients from the Kunshan factory explosion in China on 8.2	157	0.920		Burns	Extreme gradient boosting Logistic regression	None	KDIGO: Creatinine and urine output
Tran et al. (71)	University of California, Davis, US	50	Accuracy	0.80–0.90	Burns	K-nearest neighbor	None	KDIGO: Creatinine and urine volume
Rashidi et al. (72)	University of California, Davis, US	50	0.87–0.92		Burns	Logistic regression K-nearest neighbor Support vector machine Random forest Deep neural networks	None	KDIGO: Creatinine and urine volume

KDIGO, Kidney Disease: Improving Global Outcomes; AKIN, Acute Kidney Injury Network; MIMIC-III, Medical Information Mart for Intensive Care III; NHS, National Health Service.

AKI is a common complication in patients with severe burns, with a reported morbidity of ~40% and a mortality rate of 73–100% (73). There are few studies on burn-associated AKI modeling using classical machine learning methods, and the representative study was completed by Tang et al. (70) in 2018, which used the XGBoost method for model training and validation and compared it with the traditional logistic regression model. The results suggested that by including the APACHE II score, percentage of third-degree burn area, 24-h post-admission rehydration, sepsis, first 24-h urine volume, SOFA score, and 48-h post-admission rehydration, the AUC value of the model constructed by the machine learning algorithm was 0.92, which was significantly higher than that of the logistic regression model of 0.875, but the limitations of the study were also obvious. Because the included patients were survivors of a dust explosion accident, the diagnosis was mainly severe burns combined with inhalation injury, so the adaptability of external promotion could not be confirmed (Table 5).

Research on the construction of machine learning prediction models for AKI in burn patients has been equally pioneering, with a different emphasis and utilization of novel biomarkers than previous modeling ideas. In a study by Rashidi et al. (72) in 2020, it was first hypothesized that machine learning approaches could enhance the predictive potential of AKI biomarkers in critical care populations such as individuals with severe burns, and the results confirmed that when used in combination with other known biomarkers such as NT-probNP or sCr, machine learning could enhance the predictive ability and clinical sensitivity of NGAL, especially deep neural networks combined with NGAL; this would provide the best model sensitivity, specificity and AUC values, achieving a prediction 68.1 h in advance according to the KDIGO standard. More importantly, acceptable results have also been obtained for the external validation of diagnostically heterogeneous cohorts such as trauma patients. Another study conducted by Nam et al. (71) in 2019 published similar findings that the use of the k-nearest neighbor method increases the identification of AKI in burn patients based on sCr, NGAL, UOP, and NT-proBNP. In previous studies, there was concern that preexisting inflammation confounded the performance of biomarkers such as NGAL, thus limiting their role in AKI diagnosis, but machine learning methods can effectively overcome this problem by identifying complex diagnostic patterns masked by confounding factors, further proving and enhancing the performance of novel markers such as NGAL and providing hope for clinicians. In future research, the concept of modeling by this type of machine learning method may be given more attention, and the validation and utilization of other markers may bring us more surprises (Table 5).

AKI has long been considered a common and serious complication of acute pancreatitis (AP), with a prevalence of ~10–42% and a poor prognosis and mortality rate of 25–75% among patients with AP-AKI (74). In recent years, research on machine learning modeling of AP-AKI has been conducted. With the first relevant study conducted by Qu et al. (63) in 2020, after modeling by multiple methods, it was found that XGBoost achieved the best performance with the highest AUC value of 0.9193, in addition to excellent performance in terms of sensitivity and specificity, while screening based on modeled variables further complemented risk factors such as the APACHE II score and C-reactive protein and total bilirubin Levels. In addition, in recent years, there has been a deeper understanding of the possible mechanisms of AP-AKI, and an increasing number of basic experimental and clinical studies have shown that the inflammatory response plays a unique role in the pathophysiology of AKI. Based on this background, the modeling study by Yang et al. (64) in 2022 focused on the inclusion of cytokine variables, and the results suggested that by introducing C-reactive protein, platelet/lymphocyte percentages neutrophil/lymphocyte percentages, cystatin C and other inflammatory indicators, random forest and other learning methods achieved AUC values of 0.725–0.902, which not only proved the reliability of the model but also further verified the possible association of inflammatory responses with AP-AKI and simultaneously provided ideas and optimization directions for subsequent studies, namely, with the in-depth study of more biomarkers. Exploring the correlation between these biomarkers and the occurrence and development of AP-AKI by using machine learning or removing confounding factors and analyzing the connection between them and other inflammatory or non-inflammatory variables is very important for improving the treatment status of AP and strengthening the primary prevention of AP-AKI (Table 5).

AKI is a common complication in patients with malignancies, with an incidence of ~7.5–9.5% (75, 76). AKI associated with malignancy (MR-AKI) not only affects ongoing treatment but is also associated with lower tumor remission rates and higher mortality rates (77). MR-AKI is associated not only with advanced age and chronic comorbidities but also with tumor-specific factors such as malignant infiltration, tumor lysis syndrome, nephrotoxic drugs and contrast therapy (78, 79). In a study by Zhang et al. (65) in 2021, data from 6,846 patients with liver and gallbladder cancer were collected, and the XGBoost method was used for modeling. In internal validation, the AUC values of the liver cancer and gallbladder cancer models were 0.822 and 0.850, respectively. In the screening of modeling variables, it was found that both sCr and eGFR contributed more than 20% to the model gain, while liver cancer treatment was found to rank third in the ability of modeling predictors of liver cancer, which further supports the impact of partial hepatectomy or liver transplantation on AKI. In the study by Li et al. (68) in 2020, 6,459 participants with gastrointestinal cancers with a high incidence in the Chinese cancer spectrum, including esophageal, gastric and colon cancers, were recruited. Variable selection was first conducted by the GLASSO method to simplify the complexity of variables and avoid overfitting and misclassification, and then modeling was performed using various methods. Finally, the efficiency of the Bayesian network was proven to be the best, with AUC values of 0.823 and 0.790 in internal and external validation, respectively. Meanwhile, the Bayesian network gave explanations for the probabilistic dependencies among the modeled variables according to the characteristics of its own algorithms; however, this relationship did not represent a causal relationship. In addition, the study did not include recognized important clinical variables, such as infection and nephrotoxic drugs, which obscured the relationship between unknown variables and AKI to some extent. In the same year, Li et al. (69) also conducted a similar study on patients with hematologic neoplasms, covering lymphoma, leukemia, and multiple myeloma among 2,395 patients, again using the GLASSO method for variable selection and subsequently achieving an AUC value of 0.835 using the Bayesian network method. The shortcomings of the two studies are consistent, but the insights for the diagnosis and treatment of oncological diseases are profound, the models in the studies not only achieved clinical detection of MR-AKI earlier than the KDIGO criteria by machine learning methods but also further elaborated the advantages of the Bayesian network to visualize and graphically display and explain complex dependencies between variables, using solid tumors or hematologic tumors as representatives (Table 5).

The model type of the above three studies was classical, but similar studies have improved the model type. For example, Scanlon et al. (66) in 2020 only predicted the risk of AKI in the next 30 days based on the blood results of tumor patients, and the random forest method predicted AKI 30 days and 1 day earlier with AUC values of 0.881 and 0.947, respectively. Moreover, a prospective study found that ~60% of AKI cases can be detected 30 days before the onset of the disease. The study was characterized by an extended prediction range and the discarding of a large amount of non-therapeutic information to ensure that the model could be used in outpatient clinics. During the 7–28 day-chemotherapy cycle of tumor patients, clinicians have more opportunities to receive predictive alerts based on routine blood test results. Another study conducted by Park et al. (67) in 2018 developed a more applicable AKI prediction model, which relaxed the limitations of existing methods and made full use of irregular and heterogeneous data to learn and train the model. The study included a total dataset of 21,422 cancer patients. Multiple modeling methods were used to predict AKI events within 14 days with an accuracy of 0.7892, a recall rate of 0.7506, and an F value of 0.7576. The purpose of this study was to construct a machine learning model using non-intensive or irregular measurement results of non-ICU patients. First, the maximum SCr value within 14 days was predicted and then used to predict the occurrence and severity of AKI. This extended model can be applied to different clinical populations, enabling AKI prevention and better clinical decision-making for cancer patients in more diverse environments (Table 5).

### 4.5. Predictive models for AKI associated with specific nephrotoxin exposures

Contrast-related operations are currently an important approach to clinical diagnosis and treatment, including imaging enhancement examination, arteriovenous embolization, and cardiovascular intervention treatment, and AKI, one of its major comorbidities, has been receiving much attention, especially regarding the construction of machine learning prediction models for AKI associated with percutaneous coronary intervention (PCI). In 2018, Huang et al. (80) conducted the first machine learning modeling study, whose included case information was the original data developed by the NCDR-CathPCI scoring system, and multiple models were constructed and comparatively evaluated by using the same amount of data and original variables, different variable selection patterns, different candidate variable preprocessing strategies, and different modeling methods. The results suggested that the best model was constructed by using all available candidate variables in their original form, alignment-based variable selection, and XGBoost computational methods, with a wider prediction range and stronger risk stratification than the currently widely accepted NCDR-CathPCI scoring system; this comparison had a statistically significant difference, although the performance improvement was weak. In the latest study by Niimi et al. (81) in 2022, their modeling data sources were consistent with those of Huang et al. (80). They also used XGBoost and logistic regression methods to model separately. In addition to AKI events, bleeding and in-hospital mortality were also included in the endpoint results. The results suggested that the XGBoost model modestly improved the discrimination of AKI events with an AUC value of 0.84, which was significantly higher than that of the traditional logistic regression model, further supporting the conclusion of Huang et al. (80). However, the optimal modeling method is not fixed, and it is related to a variety of factors. For example, in a study by Sun et al. (82) in 2020 comparing the model performance of decision trees, support vector machines, and random forests, the results suggested that random forests eventually achieved an AUC value of 0.82 by using variables such as neutrophil percentage, age, and free triiodothyronine (Table 6).

**Table 6:** Prediction model for AKI associated with specific nephrotoxin exposure.

**Reference**	**Modeling data sources**	**Data volume**	**Model performance AUC value**		**Type of exposure**	**Machine learning methods**	**Model explanation**	**Diagnostic criteria**
Huang et al. (80)	American College of Cardiology (ACC) National Cardiovascular Data Registry (NCDR)	947,091	0.752		Contrast agents	Extreme gradient boosting	None	AKIN: Creatinine
Sun et al. (82)	Changzhou No. 2 People's Hospital of Nanjing Medical University, China	1,459	0.85		Contrast agents	Decision tree Support vector machines Random forest nearest neighbor Naive Bayes Gradient boosting machine	None	KDIGO: Creatinine and urine volume
Niimi et al. (81)	Japan Cardiovascular Database-Keio Interhospital Cardiovascular Studies (JCD-KiCS)	22,958	0.838			Logistic regression Extreme gradient boosting	None	KDIGO: Creatinine
Huang et al. (83)	American College of Cardiology (ACC) National Cardiovascular Data Registry (NCDR)	2,076,694	Contrast agent 0.3 mg	0.777	Contrast agents	Generalized additive model	None	Modified definition
			Contrast agent 0.5 mg	0.839				
			Contrast agent 1.0 mg	0.870				
Ibrahim et al. (84)	Massachusetts General Hospital in Boston, Massachusetts, USA	889	0.79		Contrast agents	LASSO regression	None	KDIGO: Creatinine
Okawa et al. (85)	Fujita Health University Hospital, Japan	1,014	0.76		Cisplatin	Neural networks Gradient boosting decision tree	None	KDIGO: Creatinine
Yang et al. (86)	Center for Medicare and Medicaid Services, US	17,694	0.72		SGLT2 inhibitors	Random forest Resilient network LASSO regression	None	Unknown

KDIGO, Kidney Disease: Improving Global Outcomes; AKIN, Acute Kidney Injury Network; SGLT2, sodium-dependent glucose transporters 2.

In addition to these three classic machine learning approach modeling studies, some investigators have continued to expand their modeling ideas. For example, in the follow-up study by Huang et al. (83) in 2019, which aimed to develop a model to assess the relationship between the volume of contrast agents received by PCI patients and the risk of AKI, the results suggested that this correlation was non-linear and heterogeneous across patients with different baseline risks, which is difficult to achieve for traditional regression methods. With the introduction of machine learning methods, based on data from more than 1 million patients covering a wide range of baseline risks, the construction of this correlation was effectively completed using a generalized additive model by quantifying the contrast agent solvent during PCI in patients with different baseline risks to enable personalized treatment. In addition, the choice of candidate variables for modeling is not invariable; for example, all modeling studies have a bias toward static or dynamic variables. Ibrahim et al. (84) did not borrow the characteristics of the variables from the former study but focused on clinical and proteomic markers, including diabetes history, urea nitrogen/creatinine ratio, C-reactive protein and bone bridging protein, which were positively correlated with AKI risk, and CD5 and VII factors that were negatively correlated. The results of the machine learning model were equally reliable, with AUC values of 0.79–0.82 obtained after modeling with data from 889 patients. In conclusion, the current research on constructing a PCI-AKI prediction model based on machine learning methods is promising, but given the widespread implementation of PCI worldwide, contrast-induced AKI remains an inescapable clinical challenge; therefore, additional research is needed to develop a model that can achieve the widespread acceptance of the NCDR-CathPCI scoring system and can truly replace the system to achieve accurate and personalized treatment to reduce the medical and economic burdens on patients with cardiovascular disease (Table 6).

Cisplatin-based chemotherapy is the first-line treatment for solid tumors such as non-small cell lung cancer, but it can easily cause renal tubular damage during excretion, leading to cisplatin-related AKI (Cis-AKI) (87, 88). To prevent Cis-AKI, aggressive or short-term hydration therapy with magnesium supplementation is clinically recommended (89). However, the incidence of Cis-AKI remains high, so early detection and prediction of Cis-AKI is essential for the management of patients treated with cisplatin for chemotherapy. According to our database search, there is only one report published by Okawa in 2021 that introduced the efficacy of the Cis-AKI machine learning prediction model, which included 1,014 oncology patients receiving cisplatin as first-line chemotherapy and excluded cases treated with angiography or intra-arterial injection of cisplatin during chemotherapy. Two methods, a neural network and gradient boosting decision tree, were used to establish models by age group. The results suggested that the models showed the highest performance among patients aged ≥75 years with an AUC value of 0.78, while in the ranking of the contributions of model prediction variables, serum albumin level, body surface area and maximum daily dose of cisplatin contributed the most to the prediction of the model. Based on the specific values of the parameters, it was shown that high-dose cisplatin (100–120 mg/day) and hypoalbuminemia (1.30–3.10 mg/dL) were risk factors for Cis-AKI in all patients. The study, despite some shortcomings, is pioneering for oncology patients, especially those treated with nephrotoxic chemotherapeutic agents such as cisplatin, and the reduction in chemotherapy-related complications is extremely critical to improve the survival rate of oncology patients. It is believed that more similar studies will be conducted in the future, which will bring more benefits for the control of side effects from AKI-related drugs such as cisplatin or immune checkpoint inhibitors (85) (Table 6).

Sodium-glucose cotransporter two inhibitors (SGLT2s) are a new oral hypoglycemic agent with positive cardiovascular protective effects but controversial effects on renal function, and a meta-analysis concluded that SGLT2s not only reduce the progression of chronic kidney disease but also have a preventive effect on AKI. (90). However, several studies, including a warning from the US Food and Drug Administration (FDA) (https://www.fda.gov/Drugs/DrugSafety/ucm505860.htm), have concluded that SGLT2s can contribute to the development of AKI by affecting patients' blood volume and inducing renal medullary damage (91, 92). Therefore, Yang et al. (86) in 2022 used a machine learning approach to establish a model to predict the risk of AKI in diabetic patients receiving SGLT2s for the first time. Based on the source data of 17,649 patients' case information, the model used the random forest method and 14 preselected variables and had an AUC value of 0.72. This study is not only representative of the predictive analysis of rare and serious adverse events, but its risk factor analysis also provides new ideas for clinical decision optimization, such as the study's conclusion that diuretics contributed most to the model and, although it cannot be determined that it has the strongest correlation with AKI, it does illustrate the impact of drug combinations on AKI (Table 6).

## 5. Conclusion

In this review study, we systematically described the research status of machine learning prediction models for AKI, summarized the data characteristics, method characteristics and result characteristics of the existing models, and provided a relatively comprehensive field summary for peer research. however, it is inevitable that the methodological introduction of some studies is still not comprehensive enough, and a small number of non-English language articles are not included in the analysis, this may have left out some of the research results. In conclusion, AKI is a worldwide health problem, and its short-term and long-term adverse effects on hospitalized patients are very obvious. However, the current challenges facing the diagnosis and treatment of AKI are still huge, including timely detection and early prediction of AKI. Based on this, future research in machine learning predictive models is likewise well directed, except to overcome the above issues, increasing the use of novel biomarkers for model training, inviting more specialized scientific and technical teams for methodological assistance, and enabling the embedding of models with health care work systems will provide greater assistance in improving the overall diagnosis and treatment status of AKI.

## Author contributions

All authors listed have made a substantial, direct, and intellectual contribution to the work and approved it for publication.
